# The Association of Organ Preservation Fluid Pathogens with Early Infection-Related Events after Kidney Transplantation

**DOI:** 10.3390/diagnostics12092248

**Published:** 2022-09-18

**Authors:** Jianming Li, Xiaojun Su, Jianyi Li, Wenrui Wu, Chenglin Wu, Penghao Guo, Kang Liao, Qian Fu, Jun Li, Longshan Liu, Changxi Wang

**Affiliations:** 1 Organ Transplant Center, The First Affiliated Hospital, Sun Yat-sen University, Guangzhou 510080, China; 2 Department of Clinical Laboratory, The First Affiliated Hospital, Sun Yat-sen University, Guangzhou 510080, China; 3 Guangdong Provincial Key Laboratory of Organ Donation and Transplant Immunology, The First Affiliated Hospital, Sun Yat-sen University, Guangzhou 510080, China; 4 Guangdong Provincial International Cooperation Base of Science and Technology (Organ Transplantation), The First Affiliated Hospital, Sun Yat-sen University, Guangzhou 510080, China

**Keywords:** preservation fluid, early infection-related events, ESKAPE, *Candida*, antimicrobial resistance, preemptive antibiotic therapy

## Abstract

(1) Background: The need to elucidate the microbial patterns in preservation fluid and explore their relationship with early infection-related events post kidney transplant and investigate antimicrobial resistance and the effects of preemptive antibiotic therapy. (2) Methods: This retrospective study analyzed the clinical data of 514 kidney transplant donors and 808 recipients from April 2015 to October 2020. Clinical data of donor and recipient characteristics, preservation fluid microbes, early infections (≤30 days), probable donor-derived infections (P-DDIs), antimicrobial resistance and preemptive antibiotic therapy was collected. (3) Results: The incidence of bloodstream (10.3% versus 5.2%, *p* = 0.006) and graft-site infections (9.7% versus 4.6%, *p* = 0.004) was significantly higher in recipients with culture-positive preservation fluid. In addition, recipients with ESKAPE pathogens or *Candida* species had a notably higher rate of bloodstream infections (14.1% versus 6.9%, *p* = 0.033) and graft-site infections (16.7% versus 3.5%, *p* < 0.01) than those with other positive pathogens. Preemptive antibiotic therapy decreased the bloodstream infection rate (11.8% versus 35.7%, *p* = 0.047) when preservation fluid was positive for ESKAPE pathogens. (4) Conclusions: Culture-positive preservation fluid has potential implications for kidney transplant recipients. ESKAPE pathogens or *Candida* species in preservation fluid as well as their antimicrobial resistance properties and non-preemptive antibiotic therapy could pose a risk of early infection-related events.

## 1. Introduction

Kidney transplant recipients are particularly prone to infections due to prolonged immunosuppression. Preservation fluid, which is critical to maintain kidney graft viability during transportation, could pose serious infection risks. Donor-derived pathogens may be present in the preservation fluid. A nationwide survey in France showed that approximately one in five kidney transplant preservation fluid samples yielded positive cultures [1], with a substantial contribution from deceased donors. Another major contributor to culture-positive preservation fluid is polymicrobial contamination occurring during organ procurement and transportation [1,2,3,4].

Donor-derived pathogens are associated with early infection-related events after kidney transplantation. Some virulent pathogens in the preservation fluid may derived from the donor, associating with early infection-related events after kidney transplantation. High-risk pathogens, including ESKAPE pathogens (*Enterococcus faecium, Staphylococcus aureus, Klebsiella pneumoniae, Acinetobacter baumannii, Pseudomonas aeruginosa, and Enterobacter species*), could lead to an increased risk of early post-transplant infections [5] and kidney transplant recipients with ESKAPE-contaminated preservation fluid are at a significantly elevated risk for probable donor-derived infections (P-DDIs) [2]. Monitoring early infection after kidney transplantation is very important in reducing infections but underutilized [6] and testing of preservation fluid is potentially an important method for monitoring the risk of early infection. Therefore, more evidence is needed to prove the relationship between preservation fluid and early infection-related events after kidney transplantation.

Further complicating the issue of preservation fluid is the possible presence of multidrug-resistant organisms as the causative microbes [7]. However, antimicrobial resistance of microbes in kidney transplant preservation fluid have rarely been investigated. Due to prolonged antimicrobial treatment in the intensive care unit and the use of large amount of antibiotics or antifungal drugs before organ procurement, donors may carry drug-resistant pathogens that could be transmitted to the recipients through organ preservation fluid [8]. ESKAPE pathogens in kidney or other solid organ transplant recipients are possibly antimicrobial-resistant and could predict an adverse clinical outcome of infections and DDIs [9,10,11]. However, few studies are available on antimicrobial resistance patterns of preservation fluid pathogens in kidney transplant [1,12]. In recent years, though many investigations have suggested that culture-positive preservation fluid is associated with infections in post-transplant recipients [2,4,13,14,15,16], it remains inconclusive whether targeted antimicrobial treatment against preservation fluid pathogens should be recommended [3]. Similarly, the benefit of preemptive antibiotic therapy against potential pathogens in preservation fluid is still unknown and needs further investigation.

In this retrospective study, we aimed to examine the microbial profile of preservation fluid and its association with early infection-related events post kidney transplant. We further sought to investigate the risk levels of different pathogens. Finally, we discussed the antimicrobial resistance of preservation fluid pathogens and explored the effect of preemptive antibiotic therapy on early infection-related events.

## 2. Materials and Methods

### 2.1. Study Population

Patients who underwent kidney transplantation from April 2015 to October 2020 at The First Affiliated Hospital of Sun Yat-sen University, China were eligible for this study. Those without complete microbe data for preservation fluid cultures and living donor kidney transplants were excluded.

### 2.2. Microbial Culture

Graft preservation fluid consisting of hyperosmotic citrate purine solution (S400, Shanghai, China) was used for graft perfusion during organ procurement and storage during graft transportation. Preservation fluid samples (10 mL each) were collected from the kidney storage bag before the back-table kidneys preparation. Each sample was inoculated into a blood culture bottle under aseptic conditions. Identification and antimicrobial susceptibility test were performed if there was microbial growth during incubation. A final report of sterile growth was recorded if there was no microbial growth after 7 days of incubation. Any microbial growth was recorded. Samples of preservation fluid for microbial assessment were processed using the Bact/Alert 3D system (bio Merieux, Marcy l’Etoile, France). We performed Antimicrobial Susceptibility Testing (AST) with the VITEK^®^ 2 system according to the manufacturer’s instructions, using the software version 8.01 and the AST-N334, AST-N335, AST-P639 cards for Gram-negative bacteria, staphylococci, enterococci and streptococci, respectively.

### 2.3. Early Infection-Related Events, Pathogen-Related Items and Preemptive Antibiotic Therapy

Early infection-related events included early infection events and P-DDIs. Early infection events [17], pathogens, and antimicrobial resistance during the first 30 days post-transplant were recorded. A graft-site infection was defined as recipients having positive microbe results (excluding possible contamination of colonizing bacteria) in peri-kidney allograft fluid collections. Bloodstream infection, including central and non-central line-associated bloodstream infection, was defined as recipients having positive microbial results in the bloodstream [18]. Wound infection was recorded when pain or tenderness, localized swelling, erythema or heat incision was found and later a positive pathogen result was detected from aseptically isolated sample of incision wound [19]. Urinary tract infection, pneumonia and infectious diarrhea were defined according to the Centers for Disease Control and Prevention/National Healthcare Safety Network guidelines [20,21,22]. We used the term of probable donor derived infections (P-DDIs) in the current study according to the definitions for imputability of donor origin infectious disease transmissions [23]. P-DDIs were diagnosed when identical microbial species and antibiotic susceptibility patterns were observed between the isolates from the donor and the recipient, and the recipient exhibited relevant symptoms [24].

Culture-positive preservation fluid was defined as growth of any pathogen in the preservation fluid. Coagulase-negative staphylococci was categorized according to the Institute of Medical Microbiology guidelines [25]. Multidrug resistance (MDR) was defined as acquired resistance to at least 1 agent in 3 or more antimicrobial categories and extensive drug resistance (XDR) was defined as acquired resistance to all but two antimicrobial categories [26]. Preemptive antibiotic therapy referred to an immediate post-transplant targeted antibiotic or antifungal treatment against the isolates of culture-positive preservation fluid without any clinical signs of active infection in the recipient [27]. 

### 2.4. Infection Prophylaxis and Immunosuppression

All recipients received empirical perioperative antibacterial treatment, with cephalosporins prior to 31 December 2018, and carbapenems afterwards. Echinocandins (Caspofungin or Micafungin) was given to recipients at high risk for fungal infection. Linezolid was given to recipients at high risk of Gram-positive coccus infection. Specific antibiotics or antifungal coverage were based on the discretion of transplant surgeons. The immunosuppressive protocol included induction with basiliximab or anti-thymocyte globin and a tapered dose of steroid, plus tacrolimus or cyclosporin A plus mycophenolic acid.

### 2.5. Statistical Analysis

Categorical variables were characterized by percentages and compared with chi-square tests or Fisher exact tests. According to their distribution, continuous variables were expressed as means and standard deviations (SD) or as medians and interquartile ranges (IQR); comparisons were made using the Student’s t-test or Mann–Whitney U test. Comparison among multiple groups were corrected by Bonferroni test. A 2-tailed *p* < 0.05 was considered statistically significant. All data analyses were performed using SPSS version 26.0 (IBM, Armonk, NY, USA) and GraphPad Prism 9.3.1(GraphPad Software, Inc, San Diego, CA, USA). 

## 3. Results

### 3.1. Donor and Recipient Characteristics

**Figure 1:**
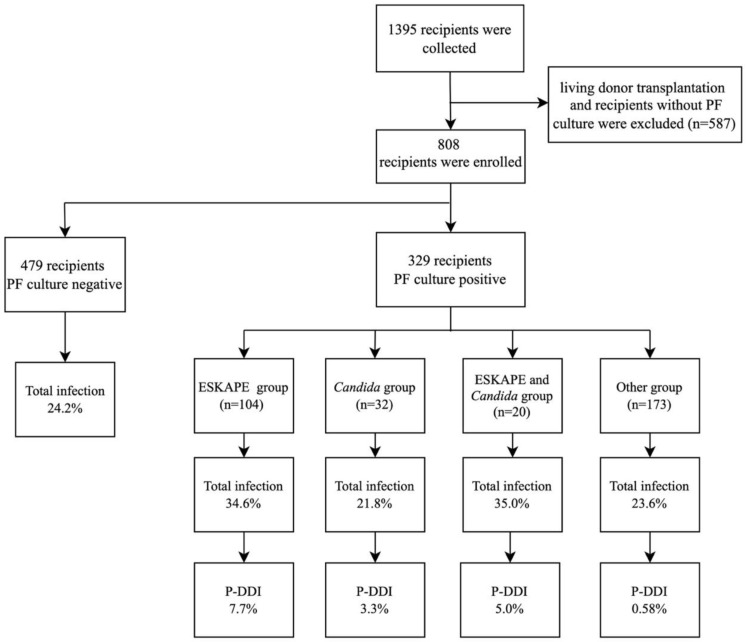
Cultures results in the preservation fluid (PF) and early infection-related events incidence rates. Recipients with simultaneous cultures of ESKAPE and other pathogens were classified in the ESKAPE group. Recipients with simultaneous cultures of *Candida* species and other pathogens were classified in the *Candida* group. Recipients with simultaneous cultures of ESKAPE and *Candida* species were classified in the ESKAPE and *Candida* group.

The study flowchart was shown in Figure 1. In total, 1395 kidney transplant recipients were reviewed, and 808 recipients who met the inclusion criteria were included in this analysis. Recipient and donor characteristics are detailed in Table 1. The mean age of the recipients was 38.6 years, and 62.9% were male. In addition, 76.9% of them were under dialysis before transplantation. The mean age of the donors was 30.2 years. 808 kidney grafts came from 514 deceased donors. Most of them were from donation after brain death (81.2%) donors. In addition, 329 recipients (40.7%) had culture-positive preservation fluid. Recipient and donor characteristics were comparable between those with culture-positive preservation fluid and those with negative result except that there was a longer ICU stay (6.1 vs. 5.0 days, *p* < 0.01) and warm ischemia time (2.8 vs. 2.3 min, *p* = 0.008) in those having a positive preservation fluid culture.

### 3.2. Microbial Profile of Kidney Graft Preservation Fluid

**Figure 2:**
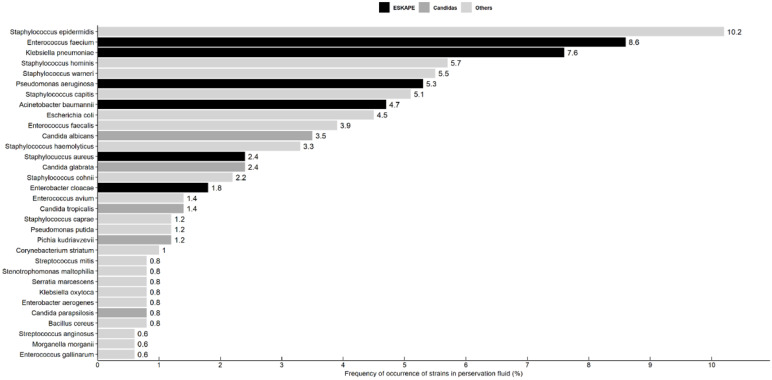
Distribution of pathogens in preservation fluid. Pathogens with a frequency of less than or equal to 0.5 percent are not shown.

A total of 490 isolates were obtained from 329 recipients with culture-positive preservation fluid. Coagulase-negative Staphylococcus accounted for 34.5% (169/490) of the isolates. ESKAPE pathogens accounted for 30.4% (149/490) of the isolates. Of these, Enterococcus faecium accounted for 28.2% (42/149). Candida species accounted for 10.6% (52/490), of which Candida albicans accounted for 32.7% (17/52) (Figure 2).

### 3.3. Incidence of Early Infection-Related Events According to Positive Versus Negative Preservation Fluid Cultures

We further analyzed the relationship between preservation fluid cultures and the incidence of early infection-related events after kidney transplantation. Ninety-one recipients with culture-positive preservation fluid (27.7%) had at least one episode of early infections while the percent of those with negative result is 24.2% (*p* = 0.271). Twelve recipients (1.5%) were diagnosed as P-DDIs, which accounted for 3.7% of recipients with culture-positive preservation fluid. The incidence of bloodstream infections was significantly higher in recipients with culture-positive preservation fluid than those with negative result (10.3% versus 5.2%, *p* = 0.006). Recipients with culture-positive preservation fluid also had a notably higher rate of graft-site infections (9.7% versus 4.6%, *p* = 0.004). A higher incidence of wound infections was observed in recipients with culture-positive preservation fluid, though there was no statistical difference between the two groups (2.7% vs 1.0%, *p* = 0.070). No significant difference was observed in the incidence of pneumonia, urinary tract infections or infectious diarrhea between recipients with culture-positive preservation fluid and recipients with culture-negative preservation fluid (Table 2).

### 3.4. Early Infection-Related Events According to Microbes

Recipients among whom ESKAPE pathogens or Candida species were detected in the preservation fluid had a notably higher rate of bloodstream infections than those who with other positive pathogens (14.1% versus 6.9%, *p* = 0.033). They also had a remarkably higher rate of graft-site infections than those who were positive for other pathogens (16.7% versus 3.5%, *p* < 0.01). Though recipients who were positive for ESKAPE pathogens or Candida species had a higher rate of urinary tract infections and wound infections, no statistically significant difference was observed (Table 3). The rate of P-DDIs in recipients who had ESKAPE pathogens or Candida species detected in preservation fluid was significantly higher (6.4% versus 1.2%, *p* = 0.011). Most P-DDIs in the kidney transplant recipients were associated with ESKAPE pathogens or Candida species. Only two cases were caused by other pathogens (Enterococcus faecalis and Stenotrophomonas maltophilia) (Table 3 and Supplementary Table S1). In addition, 34.6% (9/26) of bloodstream infections and 22.7% (5/22) of graft-site infections as well as 66.7% (4/6) of wound infections and 36.4% (4/11) of urinary tract infections were eventually identified as P-DDIs caused by ESKAPE pathogens or Candida species (Table 3 and Supplementary Tables S2 and S3).

### 3.5. Antimicrobial Resistance in Preservation Fluid and Early Infection-Related Events

One hundred seventy-nine (54.4%) recipients had had MDR microbes in the preservation fluid used for their respective kidney transplant. Recipients with MDR microbes in preservation fluid accounted for 73.5% (25/34), 66.7% (6/9) and 71.9% (23/32) of those who developed bloodstream infections, wound infections and graft-site infections, respectably. In addition, among the recipients who developed P-DDIs, 83.3%(10/12) had received transplants when there were MDR microbes in the preservation fluid (Table 4). Sixty-one (18.5%) recipients had received transplants when there were XDR microbes in the preservation fluid. Recipients with XDR microbes in preservation fluid accounted for 26.5% (9/34), 44.4% (4/9) and 28.1% (9/32) of those who developed bloodstream infections, wound infections and graft-site infections, respectively. Moreover, among the recipients who developed P-DDIs, 58.3% (7/12) had received transplants when there were XDR microbes in the preservation fluid.

Similarly, we further analyzed the antimicrobial resistance of ESKAPE pathogens. MDR microbes were present in 65.3% (81/124) of recipients who had ESKAPE-contaminated preservation fluid. In addition, MDR microbes accounted for 77.8% (14/18) of bloodstream infections, all (6/6) wound infections and 75.0% (15/20) of graft-site infections in recipients who had ESKAPE-contaminated preservation fluid. Moreover, MDR microbes accounted for 88.9% (8/9) of P-DDIs in recipients who had ESKAPE-contaminated preservation fluid. In addition, 30.7% (38/124) of recipients who had ESKAPE-contaminated preservation fluid had XDR microbes in preservation fluid. Recipients who had ESKAPE-contaminated preservation fluid had XDR microbes for 6 out of 9 P-DDIs (Table 5).

### 3.6. Preemptive Antibiotic Therapy against ESKAPE or Candida and Early Infection-Related Events

**Figure 3:**
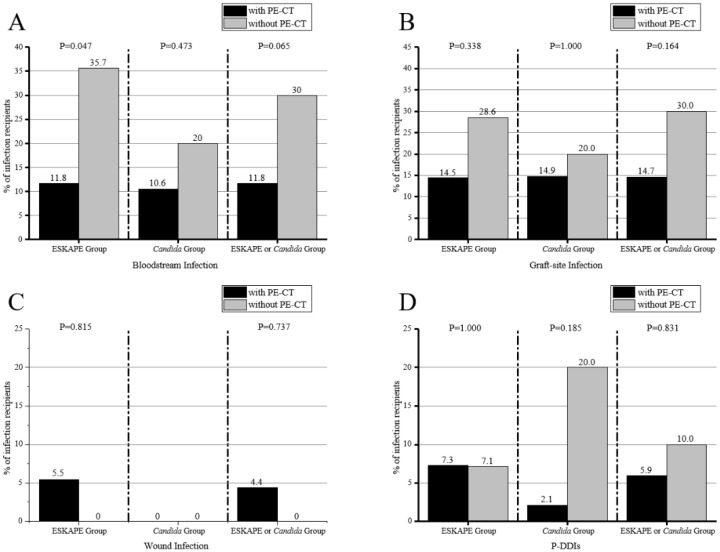
The effect of preemptive antibiotic therapy (PE-T) of preservation fluid pathogens in early infection-related events after kidney transplantation. Effect of PE-T on bloodstream infection between the ESKAPE group, *Candida* group, and ESKAPE or *Candida* group (**A**). Effect of PE-T on graft-site infection between the ESKAPE group, *Candida* group, and ESKAPE or *Candida* group (**B**). Effect of PE-T on wound infection between the ESKAPE group, *Candida* group, and ESKAPE or *Candida* group (**C**). Effect of PE-T on P-DDIs between the ESKAPE group, *Candida* group, and ESKAPE or *Candida* group (**D**).

In our study, 11.3% (14/124) and 9.6% (5/52) of recipients did not receive targeted antimicrobial treatment against ESKAPE pathogens or Candida species in preservation fluid, respectively, on a pre-emptive basis. Among recipients with ESKAPE pathogens or Candida species in preservation fluid, those who received preemptive antibiotic therapy had a lower rate of bloodstream infections, but there was no statistical difference between the two groups (11.8% vs. 30.0%, *p* = 0.065) (Figure 3). A lower incidence of graft-site infections (14.7% vs. 30.0%, *p* = 0.164) and P-DDIs (5.9% vs. 10.0%, *p* = 0.831) were observed in recipients who received preemptive antibiotic therapy than recipients who did not. However, there was also no statistical difference between the two groups. Furthermore, among recipients wtih ESKAPE-contaminated preservation fluid, those who received preemptive antibiotic therapy had a lower rate of bloodstream infections (11.8% vs. 35.7%, *p* = 0.047).

## 4. Discussion

Infections are a significant cause of morbidity and mortality after kidney transplantation [28]. DDI, although rare, once it occurs, can be associated with significant graft failure and recipients’ mortality [29]. Pathogens in the preservation fluid may be transmitted to the recipients and cause infections and even DDIs. However, the implications of preservation fluid contamination are not well established [3]. Our study examined the microbial profile of kidney transplant preservation fluid in our center and found a culture-positive rate as high as 40.7%. Unsurprisingly, incidence of bloodstream infections and graft-site infections was significantly higher in recipients with culture-positive preservation fluid than those with a negative result. Recipients who had ESKAPE pathogens or *Candida* species in the preservation fluid were significantly more likely to develop bloodstream infections, graft site infections and P-DDIs than those with other pathogens. Furthermore, preemptive antibiotic therapy with targeted antimicrobial treatment against ESKAPE pathogens or *Candida* species was beneficial to lower the incidence of early infection-related events. Notably, MDR was associated with a higher incidence of bloodstream infections and graft-site infections as well as P-DDIs. Our study demonstrated the possible impact of culture-positive preservation fluid on kidney transplant recipients and added evidence to suggest preemptive antibiotic therapy targeted antimicrobial treatment against ESKAPE pathogens or *Candida* species detected in preservation fluid.

The incidence of culture-positive preservation fluid at our center was 40.7%. Different studies report different levels of culture-positive preservation fluid incidence in solid-organ transplantation, which range from 10% up to >90% [3]. Multiple factors can affect the incidence of culture-positive preservation fluid. The main factor determining the incidence is the donor situation. Donors who have received prolonged treatment at an ICU and undergone numerous invasive procedures are at a high risk of carrying pathogens in the donated organs, resulting in a high culture-positive preservation fluid rate [8,30]. Our study found that donors with culture-positive preservation fluid had significantly longer stays in the ICU. Therefore, we suggest that such time should be minimized. Once family opinion and consent about organ transplant have been obtained, death-confirmed tests and donor procedures are carried out as early as possible. To enable timely donation procedures, ICU teams are required to manage infection and maintain organ function in potential donors. In addition, injecting samples directly into blood culture bottles and without adding antibacterial or antifungal drugs into the preservation fluid were important reason for the high positive rate in our center.

Many studies have shown that culture-positive preservation fluid is associated with the incidence of early infection-related events in kidney transplantation [4,13]. In our study, the incidence of overall early infections was higher in recipients with culture-positive preservation fluid, with a statistical difference in the incidence of bloodstream infections and graft-site infections compared with those had a negative result. Furthermore, we identified a total of 12 cases of P-DDIs. Interestingly, we found that all but one of the P-DDIs occurred in the graft site, blood circulation, surgical wound, and urinary tract, suggesting that P-DDIs occur mainly at these sites and infections occurring in these sites require extra attention.

Recipients with highly virulent pathogens isolated from the preservation fluid are more susceptible to early infection-related events after kidney transplantation [2,16]. In our study, ESKAPE pathogens or *Candida* species were associated with a higher incidence of early infection events compared to other pathogens, notably bloodstream infections, graft-site infections and P-DDIs. Yu et al. also showed a high rate of preservation fluid-related infections following contamination by ESKAPE pathogens and *Candida* species [2]. ESKAPE pathogens cause an increasing number of healthcare-associated infections with significant morbidity and mortality in solid organ transplantation recipients [11]. Up to 2/3 antimicrobial resistance rates of ESKAPE pathogens were observed in our study. The acquisition of antimicrobial resistance genes by ESKAPE pathogens has reduced the treatment options for serious infections, increased the burden of disease, and increased death rates due to treatment failure [31]. *Candida* infections can cause considerable morbidity and mortality in solid organ transplant [32]. We found that two cases of P-DDIs were caused by *Candida* species. *Candida* species can be transmitted to the recipient via the donor kidney, and it is prone to cause *Candida* arteritis, which is a possible and common manifestation of graft-site candidiasis and can pose a life-threatening situation by causing anastomotic leakage or rupture and subsequent hemorrhagic shock [33]. Consistent with our findings, a previous study suggested that *Candida* species in preservation fluid also demand attention [34].

Preservation fluid containing ESKAPE pathogens or *Candida* species supports a prediction of early infection-related events after kidney transplantation. Lacking an adequate surveillance system during donor management as well as monitoring of preservation fluid and delayed treatment are the causes of serious infections. If donor cultures are positive for the above pathogens, antimicrobial drugs should be administered prior to the donation procedure and organs procured with repeated negative cultures. In addition, routinely monitoring the culture of pathogens in preservation fluid is necessary. Timely administration of the antimicrobial drugs according to drug sensitivity results should be undertaken if these pathogens are found in the preservation fluid or recipient. Therefore, strategies for ESKAPE pathogens or *Candida* species in kidney transplant should include donor management, monitoring of preservation fluid and timely therapy of antimicrobial resistance.

Preemptive antibiotic therapy based on preservation fluid is still controversial. A recent national questionnaire survey in France found that antibiotic prophylaxis in the perioperative period of kidney transplantation is very heterogeneous in the absence of well-established guidelines [12]. A prospective study in Spain found that transmission of infection was low among recipients given preemptive antibiotic therapy for organisms isolated in preservation fluid [27]. In our study, among recipients who had ESKAPE-contaminated preservation fluid, those who received preemptive antibiotic therapy had a lower rate of bloodstream infections. In addition, among recipients with ESKAPE pathogens or *Candida* species in preservation fluid, preemptive antibiotic therapy had the effect of lower rates of bloodstream infections, graft-site infections and P-DDIs, suggesting that such pathogens in the preservation fluid should be targeted with antimicrobial treatment by prophylactic empirical antibiotic or antifungal drugs. However, Ranghino et al. found that preemptive antibiotic therapy made no difference to the rate of infections from preservation fluid related pathogens [14]. This is possibly due to the different incidence of ESKAPE-contaminated preservation fluid (18.8% vs. 30.4%). In addition, their research focused on urinary tract infections. According to our study, we considered that the analysis of preemptive antibiotic therapy should focus on bloodstream infections, graft site infections, and P-DDIs. Therefore, preemptive antibiotic therapy of virulent pathogens is necessary.

Antimicrobial resistance could increase the risk of early infection-related events after kidney transplantation. DDIs caused by MDR pathogens can have devastating consequences for organ transplant recipients [5]. We found a higher incidence of early infection-related events in recipients with MDR microbes than those without, with significantly higher rates of bloodstream infections, graft-site infections and P-DDIs. Recipients with XDR microbes also had notably higher rates of P-DDIs. These issues have rarely been addressed by other investigators. MDR infections can be acquired by the recipient through the donor’s graft in the setting of organ transplantation. However, due to lack of a well-established donor and preservation fluid monitoring procedure, transmission of MDR pathogens through the donor is difficult to diagnose, leading to delayed diagnosis, treatments, and high mortality [35]. The drug resistance rate of our preservation fluid was high. Most donors had received various invasive procedures and therefore were susceptible to pathogen infections. Meanwhile, with prolonged treatment in the ICUs and extensive use of antibiotics or antifungal drug, pathogens with antibiotic escape properties may emerge [36,37]. It is therefore important to strengthen donor management and monitoring of drug resistance of pathogens in the preservation fluid.

There are several limitations in this study. Firstly, it was a retrospective study with a single-center design. In addition, DDIs events were not eligible for detection, so we used the concept of P-DDIs instead. Lastly, the sample size of wound infection recipients was relatively small, which may lead to unreliability of the results presented for preemptive antibiotic therapy analysis about wound infections.

## 5. Conclusions

There is a potential impact of culture-positive preservation fluid on kidney transplant recipients. ESKAPE pathogens or *Candida* species in preservation fluid as well as their antimicrobial resistance properties and non-preemptive antibiotic therapy could pose a risk of early infection-related events. Therefore, we suggest that preemptive antibiotic therapy should always be used when ESKAPE or *Candida* pathogens are detected in preservation fluid, especially if they are determined as having resistance to antimicrobial treatments.

## Figures and Tables

**Table 1:** Donor and recipient characteristics.

		Preservation Fluid Cultures		
	All (N = 808)	Positive (n = 329)	Negative (n = 479)	*p* Value ^a^
**Recipients**				
Age, years, mean ± SD	38.6 ± 17.4	39.0 ± 17.4	38.3 ± 17.4	0.401
Male, n (%)	508 (62.9)	206 (62.6)	302 (63.1)	0.900
Blood types, n (%)				0.446
O	295 (36.5)	116 (35.3)	179 (37.4)	
B	197 (24.4)	88 (26.8)	109 (22.8)	
A	241 (29.8)	99 (30.1)	142 (29.7)	
AB	75 (9.3)	26 (7.9)	49 (10.2)	
Diabetes, n (%)	40 (5.0)	14 (4.3)	26 (5.4)	0.454
Dialysis type, n (%)				0.658
Hemodialysis	415 (51.4)	174 (52.9)	241 (50.3)	
Peritoneal dialysis	206 (25.5)	84 (25.5)	122 (25.5)	
None	187 (23.1)	71 (21.6)	116 (24.2)	
DGF, n (%)	123 (15.2)	54 (16.4)	69 (14.4)	0.435
**Donors**				
Age, years, mean± SD	30.2 ± 20.4	31.3 ± 20.7	29.4 ± 20.2	0.146
Male, n (%)	566 (70.1)	233 (70.8)	333 (69.5)	0.802
ICU stay, days, mean (IQR)	5.5 (3.3)	6.1 (4.0)	5.0 (2.6)	<0.01
Cause of death, n (%)				0.131
Traumatic injuries	326 (40.4)	143 (43.5)	183 (38.2)	
Cerebrovascular accidents	218 (27.0)	88 (26.7)	130 (27.1)	
Hypoxic brain injury	38 (4.7)	15 (4.6)	23 (4.8)	
Others	164 (20.3)	67 (20.4)	97 (20.3)	
Unknown	62 (7.7)	16 (4.9)	46 (9.6)	
Donor type, n (%)				0.115
DBD	656 (81.2)	258 (78.4)	398 (83.1)	
DCD	122 (15.1)	54 (16.4)	68 (14.2)	
DBCD	30 (3.7)	17 (2.1)	13 (1.6)	
Cold ischemia time in hours, mean (IQR)	10.9 (7.0)	11.1 (7.0)	10.8 (7.0)	0.385
Warm ischemia time in mins, mean (IQR)	2.5 (0.0)	2.8 (0.0)	2.3 (1.0)	0.008
Combined transplantation, n (%)	26 (3.2)	12 (3.7)	14 (2.9)	0.566

^a^ Comparison between positive group and negative group. DBD, donation after brain death; DCD, donation after circulatory death; DBCD, donation after brain death followed by circulatory death; DGF, delayed graft function; SD, standard deviation; IQR, interquartile range.

**Table 2:** Impact of cultures outcomes in preservation fluid on early infection-related events after kidney transplantation.

	Preservation Fluid Cultures				
Infection Events	Positive (n = 329)		Negative (n = 479)		*p*-Value
Overall infections	91	27.7%	116	24.2%	0.271
Pneumonia	34	10.3%	59	12.3%	0.386
Bloodstream infections	34	10.3%	25	5.2%	0.006
Wound infections	9	2.7%	5	1.0%	0.070
Graft-site infections	32	9.7%	22	4.6%	0.004
Urinary tract infections	21	6.4%	22	4.6%	0.265
Infectious diarrhea	11	3.3%	9	1.9%	0.188
P-DDIs	12	3.7%	0	0%	-

**Table 3:** Impact of ESKAPE or *Candida* group in preservation fluid on early infection-related events.

	Microbes				
	ESKAPE or *Candida* (n = 156)		Others (n = 173)		*p*-Value
**Early infections**					
Bloodstream infection	22	14.1%	12	6.9%	0.033
Wound infection	6	3.8%	3	1.7%	0.317
Graft-site infections	26	16.7%	6	3.5%	<0.01
Urinary tract infection	11	7.1%	10	5.8%	0.638
**P-DDIs**	10	6.4%	2	1.2%	0.011
Blood circulation	5	3.2%	0	0.0%	0.023
Surgical wound	4	2.6%	0	0.0%	0.050
Graft site	9	5.8%	1	0.6%	0.016
Urinary tract	4	2.6%	0	0.0%	0.050

**Table 4:** Impact of antimicrobial resistance of pathogens on early infection-related events after kidney transplantation.

Infection Events	All	MDR (n = 179)		No MDR (n = 150)		*p*-Value	XDR (n = 61)		No XDR (n = 268)		*p*-Value
Bloodstream infection	34	25	73.5%	9	26.5%	0.018	9	26.5%	25	73.5%	0.209
Wound infection	9	6	66.7%	3	33.3%	0.517	4	44.4%	5	55.6%	0.111
Graft-site infection	32	23	71.9%	9	28.1%	0.037	9	28.1%	23	71.9%	0.142
P-DDIs	12	10	83.3%	2	16.7%	0.040	7	58.3%	5	41.7%	0.001

**Table 5:** Impact of antimicrobial resistance in ESKAPE on early infection-related events after kidney transplantation.

Infection Events	All	MDR (n = 81)		No MDR (n = 43)		*p*-Value	XDR (n = 38)		No XDR (n = 86)		*p*-Value
Bloodstream infection	18	14	77.8%	4	22.2%	0.230	7	38.9%	11	61.1%	0.412
Wound infection	6	6	100%	0	0.0%	0.092	4	66.7%	2	33.3%	0.071
Graft-site infection	20	15	75.0%	5	25.0%	0.321	7	35.0%	13	65.0%	0.645
P-DDIs	9	8	88.9%	1	11.1%	0.238	6	66.7%	3	33.7%	0.040

## Data Availability

Not applicable.

## Supplementary Materials

The following supporting information can be downloaded at: https://www.mdpi.com/article/10.3390/diagnostics12092248/s1, Table S1: Twelve cases of probable donor derived infections; Table S2: Impact of different pathogens in preservation fluid on infection-related events; Table S3: Impact of different groups in preservation fluid on probable donor-derived events.
