# Supplementary material for: The Association of Organ Preservation Fluid Pathogens with Early Infection-Related Events after Kidney Transplantation
Source: Diagnostics (Basel). 2022 Sep 18;12(9):2248. doi: 10.3390/diagnostics12092248 (PMC9497690; doi:10.3390/diagnostics12092248)
Supplement: Supplementary file 1 [file diagnostics-12-02248-s001.zip › diagnostics-1875704-supplementary.pdf]

Supplementary materials

Supplementary Table S1 Twelve cases of probable donor derived infections

Recipients	Age/Gender	Pathogens in preservation fluid	Pathogens of P-DDIs	Graft site	Blood circulation	Surgical wound	Urinary tract	Other sites
1	37/M	*Enterococcus faecium, Enterococcus faecalis, Klebsiella pneumoniae*	*Enterococcus faecium*	Yes	Yes	No	No	No
2	9/M	*Staphylocuccus aureus*	*Staphylocuccus aureus*	Yes	Yes	Yes	Yes	No
3	43/F	*Enterococcus faecium, Klebsiella pneumoniae*	*Enterococcus faecium*	Yes	Yes	Yes	No	No
4	49/F	*Acinetobacter baumannii*	*Acinetobacter baumannii*	Yes	No	Yes	Yes	No
5	59/M	*Cyberlindnera jadinii*	*Cyberlindnera jadinii*	No	Yes	No	No	No
6	40/M	*Enterococcus faecalis*	*Enterococcus faecalis*	Yes	No	No	No	No
7	50/F	*Enterococcus faecium, Leuconostoc lactis*	*Enterococcus faecium*	Yes	No	No	No	No
8	55/M	*Enterococcus faecium, Klebsiella pneumoniae*	*Enterococcus faecium, Klebsiella pneumoniae*	Yes	Yes	Yes	Yes	No
9	35/F	*Klebsiella pneumoniae*	*Klebsiella pneumoniae*	Yes	No	No	No	No
10	36/M	*Acinetobacter baumannii*	*Acinetobacter baumannii*	Yes	No	No	No	No
11	59/M	*Stenotrophomonas maltophilia*	*Stenotrophomonas maltophilia*	No	No	No	No	Pulmonary
12	57/M	*Candida albicans, Enterococcus faecium, Enterococcus faecalis*	*Candida albicans, Enterococcus faecium*	Yes	No	No	No	No

Supplementary Table S2 Impact of different pathogens in preservation fluid on infection-related events

Infection-related events	Other pathogens (n=173)		ESKAPE (n=124)		P-value [a]	*Candida* (n=32)		P-value [b]	ESKAPE or *Candida* (n=156)		P-value [c]
Bloodstream infection	12	6.9%	18	14.5%	0.198	4	12.5%	1.000	22	14.1%	0.033
Wound infection	3	1.7%	6	4.8%	1.000	0	0.0%	1.000	6	3.8%	0.317
Graft-site infection	6	3.5%	20	16.1%	0.000	6	18.8%	0.030	26	16.7%	<0.01
Urinary tract infection	10	5.8%	8	8.9%	1.000	3	9.4%	1.000	11	7.1%	0.638
P-DDIs	2	1.2%	9	7.3%	0.090	1	3.1%	1.000	10	6.4%	0.011

[a] Comparison between recipients with ESKAPE pathogens in preservation fluid and recipients with other pathogens in preservation fluid.

[b] Comparison between recipients with *Candida* species in preservation fluid and recipients with other pathogens in preservation fluid.

[c] Comparison between recipients with ESKAPE or *Candida* pathogens in preservation fluid and recipients with other pathogens in preservation fluid.

P-values are corrected by Bonferroni.

Supplementary Table S3 Impact of different groups in preservation fluid on probable donor-derived events

P-DDIs	Other pathogens (n=173)		ESKAPE (n=124)		P-value [a]	*Candida* (n=32)		P-value [b]	ESKAPE or *Candida* (n=156)		P-value [c]
Blood circulation	0	0.0%	4	3.2%	0.180	1	3.1%	1.000	5	3.2%	0.023
Surgical wound	0	0.0%	4	3.2%	0.180	-	-	-	4	2.6%	0.050
Graft-site	1	0.6%	9	7.3%	0.030	0	0.0%	1.000	9	5.8%	0.016
Urinary tract	0	0.0%	4	3.2%	0.180	-	-	-	4	2.6%	0.050

[a] Comparison between recipients with ESKAPE pathogens in preservation fluid and recipients with other pathogens in preservation fluid.

[b] Comparison between recipients with *Candida* species in preservation fluid and recipients with other pathogens in preservation fluid.

[c] Comparison between recipients with ESKAPE or *Candida* pathogens in preservation fluid and recipients with other pathogens in preservation fluid.

P-values are corrected by Bonferroni.
